# Pien Tze Huang alleviate the joint inflammation in collagen-induced arthritis mice

**DOI:** 10.1186/s13020-020-00311-3

**Published:** 2020-03-30

**Authors:** YongQi Deng, Hui Luo, Jun Shu, Haiyang Shu, Cheng Lu, Ning Zhao, Yun Geng, Xiaojuan He, Aiping Lu

**Affiliations:** 1 Institute of Basic Research in Clinical Medicine, China Academy of Chinese Medical Sciences, Beijing, China; 2 School of Life Science and Engineering, Southwest Jiaotong University, Chengdu, China; 3 Institute of Clinical Medical Science, China-Japan Friendship Hospital, Beijing, China; 4 The Second Clinical College of Guangzhou, University of Chinese Medicine, Guangzhou, China; 5 Law Sau Fai Institute for Advancing Translational Medicine in Bone and Joint Diseases, School of Chinese Medicine, Hong Kong Baptist University, Kowloon Tong, Hong Kong China; 6 Academy of Integrative Medicine, Shanghai University of Traditional Chinese Medicine, Shanghai, China

**Keywords:** Rheumatoid arthritis, Pien Tze Huang, NF-κB signaling pathway, NLRP3 inflammasome

## Abstract

**Background:**

Rheumatoid arthritis (RA) is an autoimmune disease characterized by synovitis. Pien Tze Huang (PZH) is a Chinese patent medicine with anti-inflammatory and immunomodulatory effects. However, whether PZH could be used in RA therapy is still unknown. Therefore, this study aimed to explore the therapeutic effect and the potential mechanism of PZH on collagen-induced arthritis (CIA) mice.

**Methods:**

Male DBA/1J mice were used to establish an animal model of CIA and then treated with different doses of PZH for 4 weeks. The therapeutic effect of PZH on CIA mice was evaluated by arthritis score, pathological staining, and detecting the levels of inflammatory factors in serum and joints. To investigate its possible mechanism, the activity of NF-κB signaling pathway, NLRP3 inflammasome and the level of A20 were detected.

**Results:**

The results showed that PZH could alleviate the erythema and swelling of hind paws of CIA mice, improve the pathological conditions of joint and decrease the production of IL-1β, IL-6 and IL-17 in serum and joints. Furthermore, PZH could significantly inhibit the activity of NF-κB signaling pathway and NLRP3 inflammasome in the ankle joint of CIA mice compared with the model group. It also increased the level of A20 in the ankle joint of CIA mice.

**Conclusion:**

This study indicated that PZH could alleviate the joint inflammation of CIA mice, and the mechanism might be related to the regulation of NF-κB signaling pathway and NLRP3 inflammasome.

## Background

Rheumatoid arthritis (RA) is an inflammatory disease characterized by extensive synovial hyperplasia and inflammatory cell infiltration, which finally cause bone damage and functional joint disability [1]. Pro-inflammatory cytokines such as IL-1β, IL-6 and IL-17 produced by synoviocytes and infiltrating immune cells are highly associated with the pathogenesis of RA [2]. They could activate other inflammatory mediators like INF-γ, iNOS and COX-2, synergistically enhanced synovial inflammation, ultimately leading to joint damage [3]. Many signaling pathways have been proved to involve in regulating the production of pro-inflammatory cytokines in RA. Besides classical NF-κB signaling pathway, the NOD-like receptor family pyrin domain-containing 3 (NLRP3) inflammasome in RA pathological progress gradually obtained concerns [4, 5]. The activation of NLRP3 inflammasome influenced not only the expression of IL-1β and IL-18, but also the activation of NF-κB signaling pathway [6, 7]. The transmission of these inflammatory signals also indicated the complexity of RA pathological process.

Clinical drugs for RA include non-steroidal anti-inflammatory drugs (NSAIDs), glucocorticoids and several biological agents, which have varying degrees of efficacy and side effects [8]. Therefore, safe and effective drugs are still urgently needed in RA treatment. Traditional Chinese medicines (TCM) are the treasure house for new drug discovery. Some TCM have been used to treat RA patients in clinic with good efficacy, such as Wu-Tou Decoction and Yi Shen Juan Bi Pill [9, 10].

Pien Tze Huang (PZH), a well‑known traditional Chinese formula, consists of *Notoginseng Radix et Rhizoma* (Tianqi or Sanqi), *calculus bovis* (Niuhuang or ox’s gallstone), Shedan (snake’s gall) and musk [11]. It has been used in China and Southeast Asia for centuries as a folk remedy for various inflammation related diseases, such as hepatitis. It was proved that PZH exerted anti-inflammatory effect by regulating the percentage of Th1 and Th17 cells, reducing the expression of IL-17, IL-23 and IFN-γ and regulating NF-κB and STAT signaling pathways in inflammatory model animals [11, 12]. Recently, some medicinal components of *Notoginseng Radix et Rhizoma* have been reported to have remarkable effect in treating mice or rats with collagen-induced arthritis (CIA), a classical RA animal model [13, 14]. In addition, ginsenoside Rg1 and ginsenoside metabolite compound K could markedly regulate NLRP3 inflammasome and NF-κB nuclear translocation [14, 15]. Based on these studies, we hypothesized that PZH might have therapeutic effect on CIA mice. Therefore, this research aimed to explore the therapeutic potential and possible mechanism of PZH on CIA mice.

## Methods

### Drugs

PZH was produced by Zhangzhou Pien Tze Huang Pharmaceutical Co., Ltd., (Zhangzhou, China; FDA approval no. Z35020243). The drug samples were identified and characterized by HPLC–MS/TOF as our previous publication [12]. Stock solution of PZH was prepared by dissolving the PZH powder in purified water and the sample was fully blended again prior to use.

### Animals

Male 6–8 week old DBA/1J mice were obtained from Beijing Vital River Laboratories (Beijing, China). The mice were fed with food and water ad libitum, and then were allowed to acclimatize themselves for 1 week before the initiation of experiment. All protocols used here received approval from the Research Ethics Committee of Institute of Basic Theory of Chinese Medicine, China Academy of Chinese Medical Sciences.

### Induction of CIA

Bovine type II collagen (CII, 2 mg/mL; Chondrex, Redmond, WA, USA) was diluted in an equal volume of complete Freund’s adjuvant (Chondrex). Mice were first immunized with 100 μL emulsion containing 100 μg CII via intradermal injection into the tail and followed by a booster immunization on day 21 [16]. The onset of CIA could be observed between day 28 and day 32 after the first immunization.

### Treatment

We divided the mice of successful induction of CIA model into six groups (six mice per group), which were normal group, model group, PZH low dose group (PZH-L, 0.078 g/kg/day), PZH middle dose group (PZH-M, 0.234 g/kg/day, equal to the clinical dose), PZH high dose group (PZH-H, 0.702 g/kg/day), and methotrexate group (MTX, 0.3 mg/kg/day, set as previously described [17]). Purified water (normal group, model group), MTX and PZH were intragastrically administered every day at a volume of 1 mL/100 g for 4 weeks.

### Arthritis score

The arthritis score was measured twice a week. Arthritis score for each ankle joint was recorded by the same observers, who were blind to the treatment received by animals as previously described. Scoring was performed with a 0–4 scale, the arthritis scores were performed as: 0 = no change; 1 = paw with mild swelling at single limb; 2 = more than one paw with swelling; 3 = all paw with swelling and obvious erythema; and 4 = whole paw with severe swelling. The maximum score of each mouse was 16 [18].

### ELISA

Samples were prepared from the mice of serum. The levels of IL-1β, IL-6 and IL-17 in serum of mice were determined by ELISA using commercial kits (Invitrogen, Carlsbad, CA, USA) according to the manufacturer’s protocol.

### Blood biochemical determination

Alanine aminotransferase (ALT), aspartate aminotransferase (AST), creatinine (CREA) and urea (UREA) in serum of mice were tested with blood biochemical commercial kits by a Japan’s Hitachi 7160 automatic biochemical analyzer.

### Histopathology

The sections (3 μm) were prepared and stained with hematoxylin and eosin (H&E). Histopathological characteristics were assessed and scored under blinded conditions according to the following system: 0, no detectable change; 1, minor focal infiltrate; 2, moderate infiltrate; 3, severe infiltrate but no pannus or erosion of cartilage; and 4, very severe infiltrate plus pannus or cartilage erosion and fibrosis [19]. The scoring was performed blindly by three independent observers.

### Immunohistochemistry

The sections were incubated respectively with anti-IL-1β (dilution 1:1000, Abcam, Cambridge, UK), anti-IL-6 (dilution 1:1000, Abcam, Cambridge, UK), anti-IL-17 (dilution 1:200, Abcam, Cambridge, UK) and anti-A20 (dilution 1:200, Abcam, Cambridge, UK) overnight at 4 °C, followed by hatching with biotinylated horse radish peroxidase-conjugated secondary antibody for 30 min at room temperature (ZSGB-BIO, Beijing, China). Peroxidase staining was developed with DAB Kit (Cell Signaling Technology, Danvers, MA, USA). Later, sections were subjected to counterstain with hematoxylin (Leagene, Beijing, China). In the negative control groups, PBS buffer was used instead of the primary antibodies. Images were captured by LEICA DM6000B with LEICA DFC300 FX at a magnification of 400×. Semi-quantitative analysis was performed as previously described [20].

### Western blotting

The ankle joints were lysed by RIPA Lysis Buffer (Beyotime, Shanghai, China), which involved PMSF (Beyotime, Shanghai, China). According to the manufacturer’s instructions, protein samples in RIPA buffer were quantified with the Micro BCA protein assay kit (Beyotime, Shanghai, China). Each sample was used for electrophoresis with 10% sodium dodecyl sulfate–polyacrylamide gels and then transferred to PVDF membranes. The PVDF membranes were blocked with 3% bovine serum albumin in Tris buffered saline-Tween 20 (TBST) buffer for 30 min at room temperature. The blots were incubated with anti-A20, anti-P65, anti-P-P65, anti-IκBα, anti-NLRP3, anti-ASC and anti-caspase-1 antibody at 1:1000 overnight at 4 °C and with horse radish peroxidase-conjugated secondary antibody (Abcam, Cambridge, UK) for 60 min at room temperature. Blots were scanned and analyzed with Image J software.

### Statistical analysis

All of the data were analyzed using GraphPad Prism 6 software and were expressed as the mean ± standard deviation (SD) using one-way ANOVA. Difference with P-value < 0.05 was considered significant.

## Results

### PZH ameliorated the symptoms of CIA mice

**Fig. 1:**
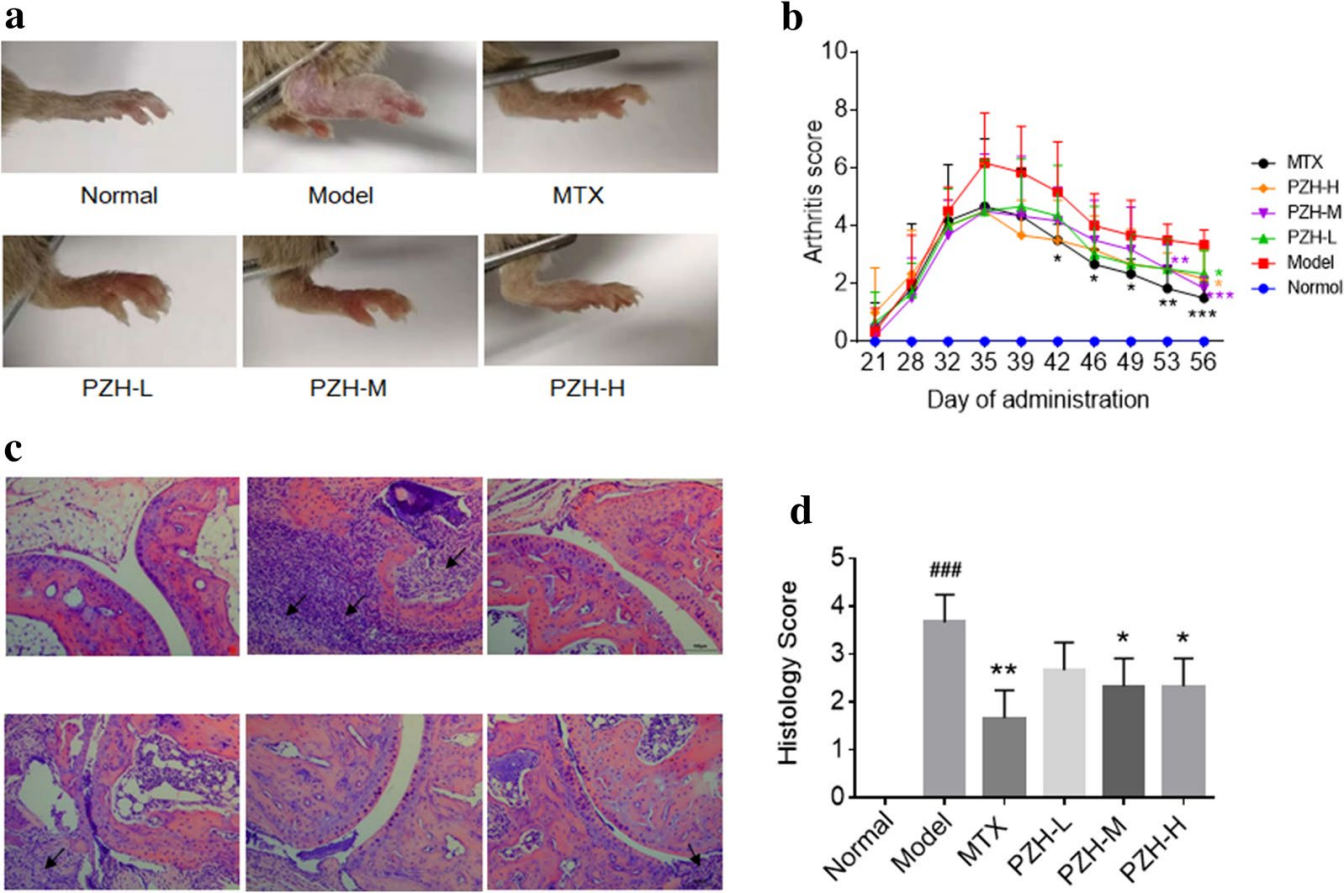
PZH ameliorated the symptoms of CIA mice. Mice were orally administered with PZH-L (0.078 g/kg/day), PZH-M (0.234 g/kg/day), PZH-H (0.702 g/kg/day), MTX (0.3 mg/kg/day) or pure water (both Normal group and Model group) for 28 days. **a** Representative photograph of ankle joint from each group. **b** Arthritis score of each group. **c** Representative pathological sections of the ankle joint by H&E staining (×200, scale bars = 100 μm). The black arrow indicates the inflammatory site. **d** Histology score of each group. n = 6 each group. ^###^P < 0.001, vs. normal group, *P < 0.05, **P < 0.01, ***P < 0.001, vs. model group

To investigate the effect of PZH on arthritis, different dose of PZH were orally administered to the CIA mice for 4 consecutive weeks, and MTX was used as a positive control. The erythema and swelling of the hind paws in CIA mice could be markedly observed, while PZH treatment ameliorated the arthritis symptoms, inhibited the swelling and erythema of the hind paws in CIA mice (Fig. 1a). Consistently, arthritis scores in PZH-treated CIA mice were significantly lower than those in model group (Fig. 1b). Histologic evaluation of the ankle joints revealed the inflammatory cells infiltration, synovial hyperplasia and joint destruction in model group. Whereas, PZH treatment could alleviate those histopathological changes (Fig. 1c, d).

### PZH decreased the production of cytokines in serum and joints of CIA mice

**Fig. 2:**
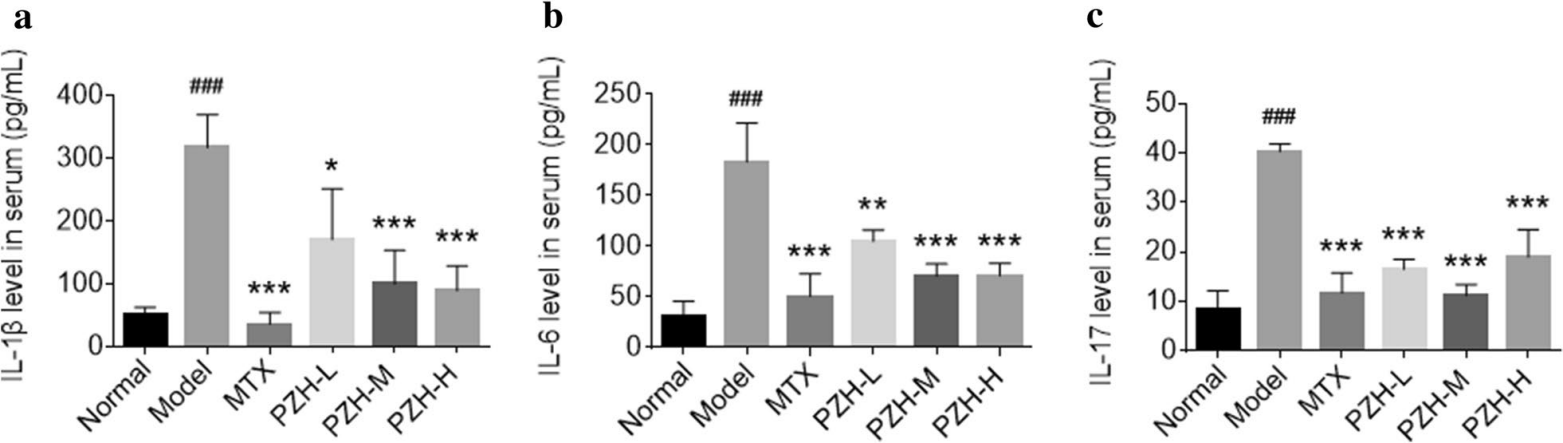
PZH suppressed the production of pro-inflammatory cytokines in serum of CIA mice. The levels of IL-1β **a**, IL-6 **b** and IL-17 **c** in serum of mice were determined by ELISA. n = 6 each group. ^###^P < 0.001, vs. normal group, *P < 0.05, **P < 0.01, ***P < 0.001, vs. model group

**Fig. 3:**
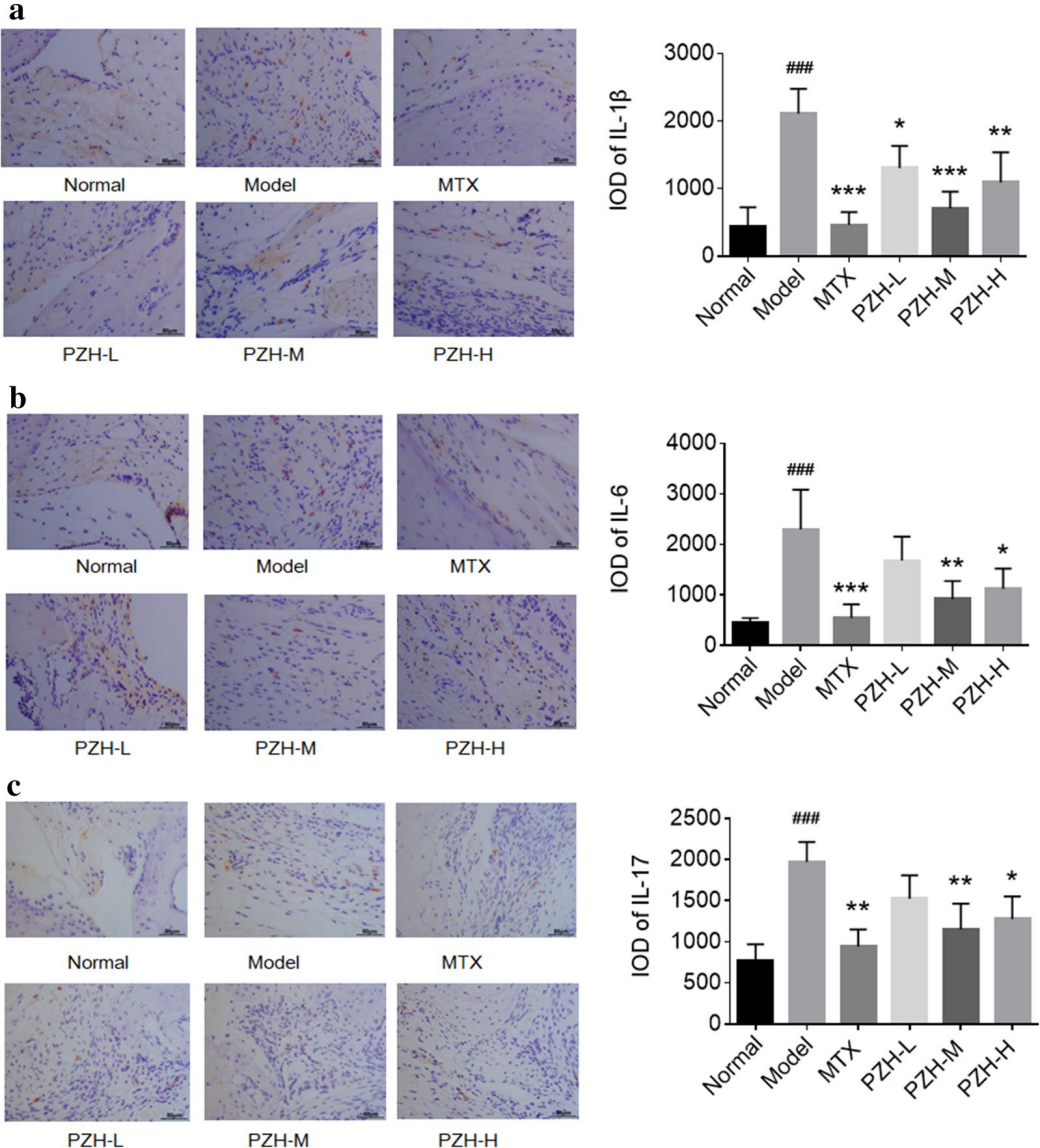
PZH decreased the levels of pro-inflammatory cytokines in the joint of CIA mice. Representative immunohistochemistry images (left) and IOD means (right) of IL-1β **a**, IL-6 **b** and IL-17 **c** in ankle joint synovium (×400, scale bars = 50 μm). n = 6 each group. ^###^P < 0.001, vs. normal group, *P < 0.05, **P < 0.01, ***P < 0.001, vs. model group

To evaluate the anti-inflammatory effect of PZH, the ELISA and immunohistochemistry analysis were used to detect the levels of pro-inflammatory cytokines in serum and synovium, respectively. The data indicated that the levels of IL-1β, IL-6 and IL-17 in serum (Fig. 2a–c) and synovium (Fig. 3a–c) of CIA mice were significantly increased in model group compared with the normal group, and decreased in groups treated with PZH compared with the model group.

### PZH inhibited the NF-κB signal pathway in the joints of CIA mice

**Fig. 4:**
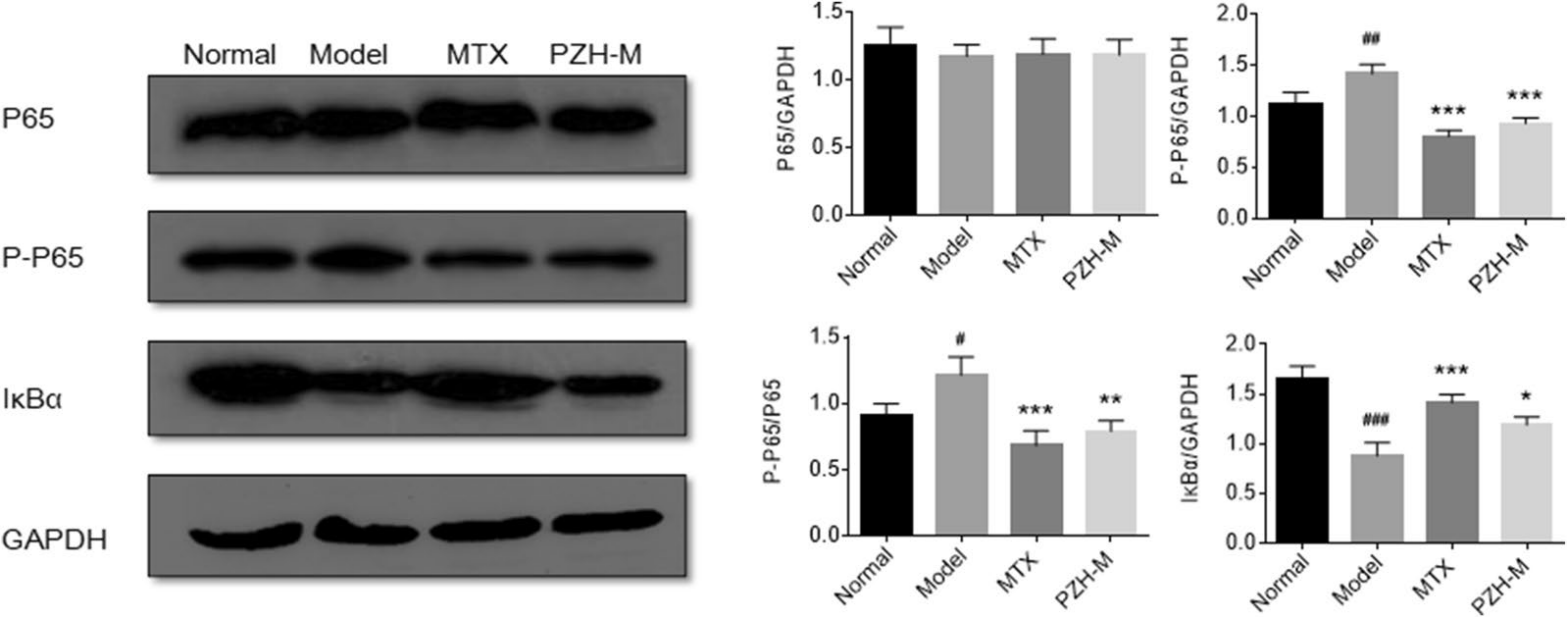
PZH inhibited NF-κB signal pathway in the joints of CIA mice. Western blot bands of P65, P-P65 and IκBα (left) and their expression relative to GAPDH or P65 (right). All experiments were performed three times. ^##^P < 0.01, ^###^P < 0.001, vs. normal group, *P < 0.05, **P < 0.01, ***P < 0.001 vs. model group

As the above results showed that PZH-M had better effect on CIA mice, we then chose PZH-M for further mechanism study. We firstly detected the expression of P65, P-P65 and IκBα in the ankle joints of mice by western blotting. As shown in Fig. 4, the level of P-P65 was significantly increased in model group compared with the normal group, and decreased in PZH treated group compared with the model group. By contrast, the level of IκBα was decreased in model group compared with the normal group, and increased in PZH treated group compared with the model group. In addition, the level of P65 had no significant difference in all groups.

### PZH suppressed the NLRP3 inflammasome in the joints of CIA mice

**Fig. 5:**
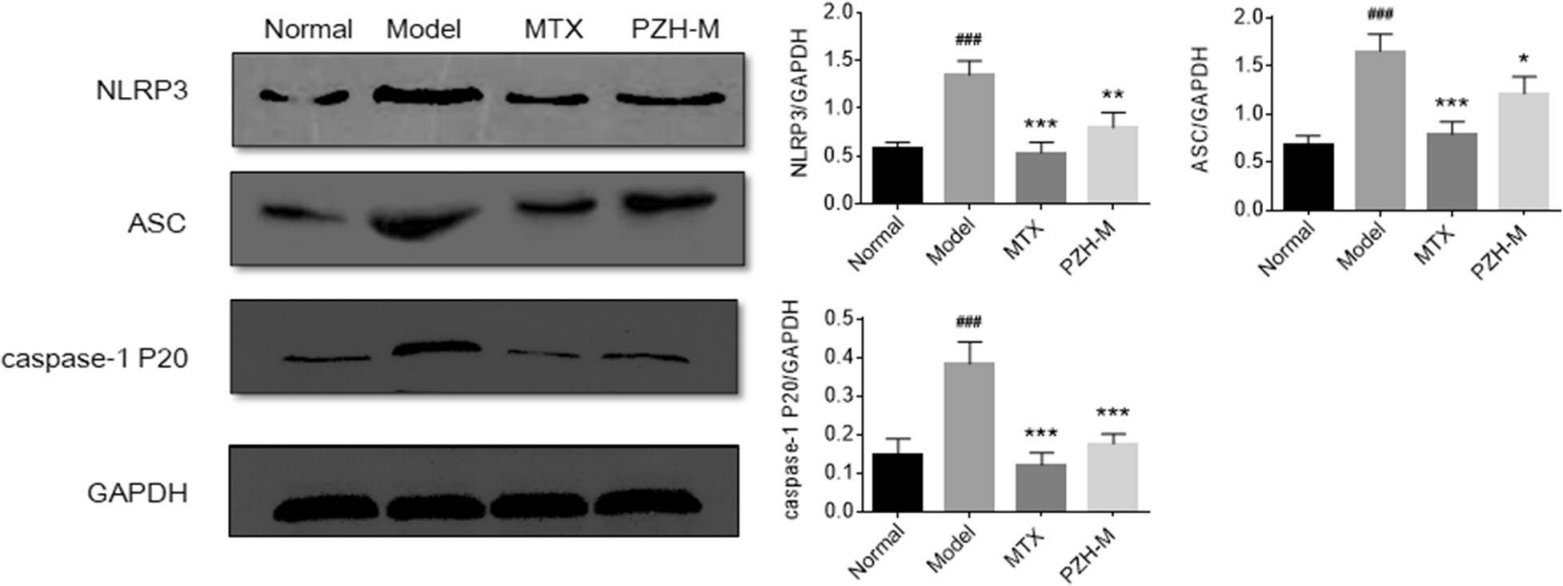
PZH inhibited NLRP3 inflammasome in the joints of CIA mice. Representative western blot bands of NLRP3, ASC and caspase-1 P20 (left) and their expression levels relative to GAPDH (right). All experiments were performed three times. ^###^P < 0.001, vs. normal group, *P < 0.05, **P < 0.01, ***P < 0.001, vs. model group

To investigate the anti-inflammatory mechanism of PZH in CIA mice, the expression of NLRP3, ASC and activated caspase-1 (caspase-1 P20) in the ankle joints of mice were detected by western blotting. The results showed that the levels of NLRP3, ASC and caspase-1 P20 was significantly increased in the model group compared with the normal group, whereas PZH treatment decreased the levels of these factors when compared to the model group (Fig. 5).

### PZH increased the production of A20 protein in the joints of CIA mice

**Fig. 6:**
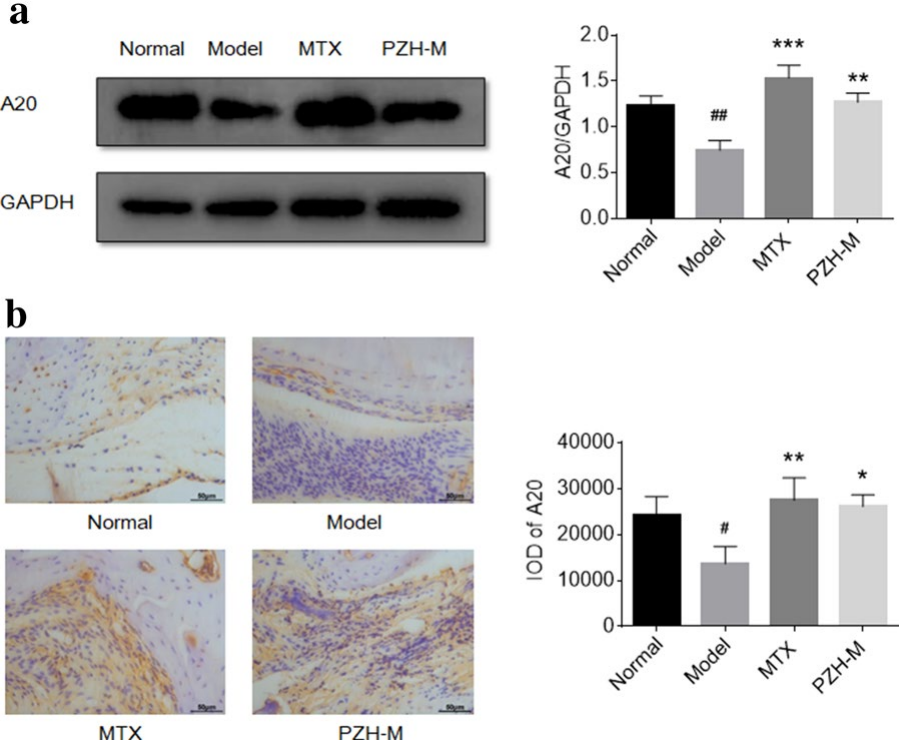
PZH suppressed the expression of A20 protein in the joint of CIA mice. **a** Representative western blot band of A20 (left) and the level of A20 relative to GAPDH (right). **b** Representative immunohistochemistry images (left) and IOD means (right) of A20 in ankle joint synovium. Original magnification ×400, scale bars = 50 μm. All experiments were performed three times. ^#^P < 0.05, ^##^P < 0.01, vs. normal group, *P < 0.05, **P < 0.01, ***P < 0.001, vs. model group

To further explore whether the anti-inflammatory mechanism of PZH was relative to the expression of A20, the western blotting and immunohistochemistry analysis were used to detect the level of the protein in the ankle joints and synovium of mice, respectively. As shown in Fig. 6, in the model group, the level of A20 significantly decreased when compared with the normal group. PZH treatment significantly increased the production of A20 when compared with the model group.

### PZH had no significant toxicity in CIA mice

**Fig. 7:**
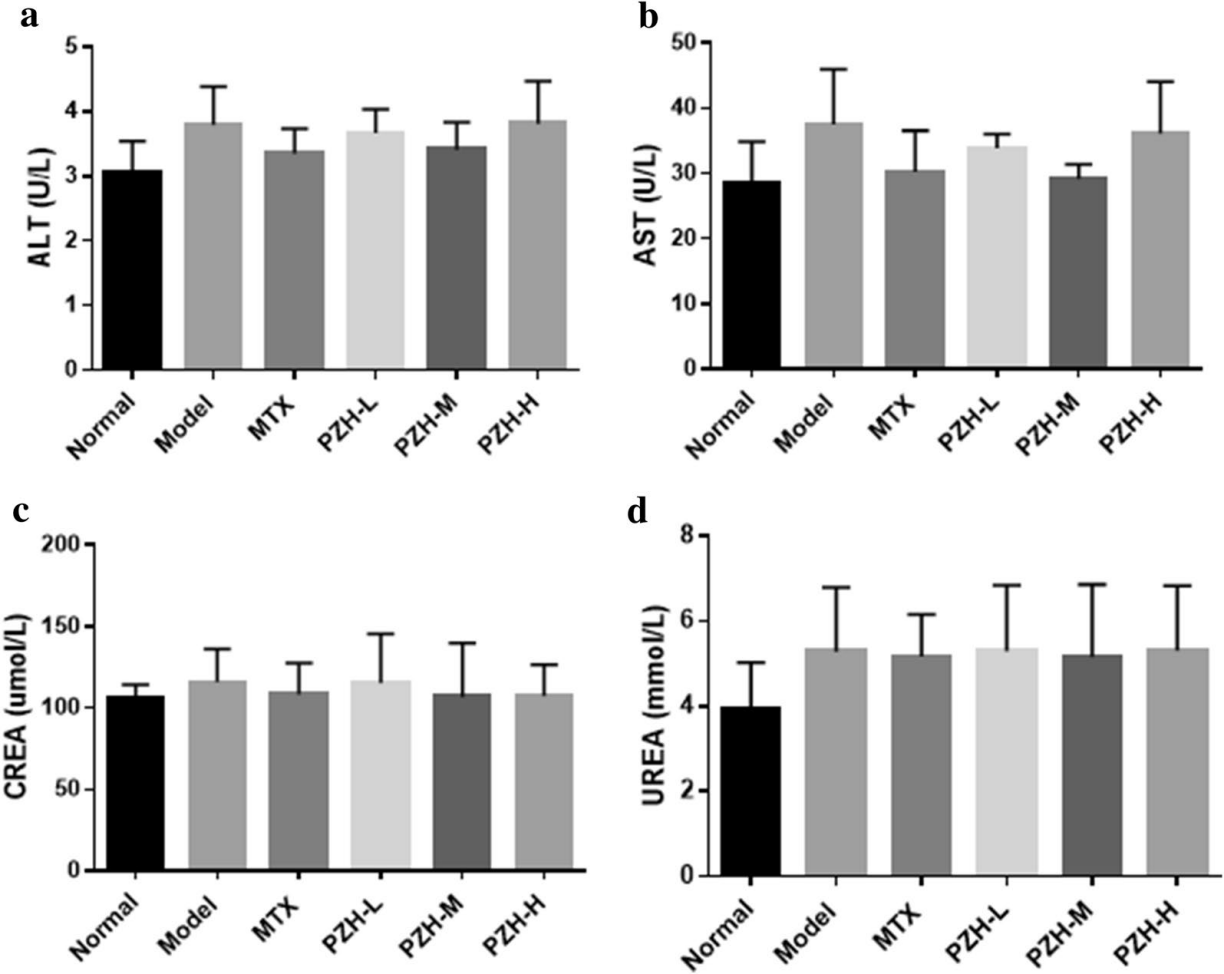
PZH had no significant toxicity in CIA mice. The levels of ALT **a**, AST **b**, CREA **c** and UREA **d** in serum of mice were shown respectively. n = 6 each group

To investigate whether PZH had toxicity in CIA mice, the levels of ALT and AST for hepatotoxicity as well as CREA and UREA for nephrotoxicity were detected. As shown in Fig. 7, PZH treatment didn’t significantly change the levels of ALT, AST, CREA and UREA.

## Discussion

PZH is an anti-inflammatory Chinese patent medicine, having therapeutic effect on hepatitis, cholecystitis and other diseases. Recently, PZH was reported to alleviate some inflammatory autoimmune diseases such as multiple sclerosis [11]. However, whether PZH could be used to treat RA was still uncertain. In this study, we immunized DBA/1J mice with bovine type II collagen to induce a CIA model and treated these CIA mice with different doses of PZH. We found that PZH could significantly relieve the erythema and swelling of the hind paws, and improve the pathological conditions in the ankle joints of CIA mice. The inflammatory cells infiltration, synovial inflammation and joint damage in CIA mice were alleviated after treatment with PZH. These results implied that PZH could be used to treat RA. Simultaneously, blood biochemical determination indicated that PZH did not show significant liver and kidney functional impairment.

The main pathological phenomenon of RA was synovitis. Long-term inflammation of synovium would accelerate the lesion and eventually aggravate joint dysfunction and malformation. Synovitis was caused by persistent inflammatory responses, which were resulted from the increased number of inflammatory cells and the increased secretion of various inflammatory mediators, such as IL-1β, IL-6 and IL-17. These cytokines could make T cells, B cells, mononuclear cells and neutrophils collect in the diseased joints, and make synoviocytes proliferation and fibrosis, stimulate synoviocytes to produce more colony stimulating factor, chemokines, proinflammatory factors and other inflammatory mediators to amplify the inflammatory response [21–23]. Currently, some inflammatory factor inhibitors have been clinically used to treat RA, including IL-6 inhibitor tocilizumab and IL-1β inhibitor anakinra [24, 25]. In this study, we detected the levels of IL-1β, IL-6, IL-17 in serum and synovium to observe the effect of PZH on CIA mice. We found that PZH could inhibit the expression of pro-inflammatory factors IL-1β, IL-6 and IL-17 in CIA mice, which also reflected the therapeutic effect of PZH on CIA mice.

Inflammatory cytokines expressed in a variety of cells and regulated by signaling pathways, which played important roles in mediation of immune and inflammatory responses in RA [26]. The most classical signal transduction pathways in RA included NF-κB signal pathway [27, 28]. Stimulation of inflammatory signals caused the continued activation of the NF-κB pathway, followed by inhibitory protein IκBα degraded, resulting to release P65 [29]. After phosphorylation, the active molecules P-P65 bound to the promoter regions of different genes, causing abnormal expressions of downstream inflammatory factors including IL-1β, IL-6 and IL-17 [30]. Previous studies have reported that the effective component of PZH, ginsenoside Rf, Rd, Rh2 and notoginsenoside R1 could regulate the activity of NF-κB signal pathway [31–34]. Our experimental results showed that PZH could inhibit the activity of NF-κB signal pathway in the ankle tissue of CIA mice, which implied that the anti-inflammatory effect of PZH might be related to the regulation of NF-κB signal pathway.

The NLRP3 inflammasome is an intracellular multimolecular complex that controls caspase-1 activity in response to various pathogen-derived factors as well as danger-associated molecules [35]. Recently, some studies indicated that NLRP3 inflammasome was involved in the pathogenesis of RA [4, 36]. The activation of NLRP3 inflammasome could be induced by toll-like receptor (TLR) and NF-κB pathway [37]. NLRP3 recruited and activated pro-caspase-1 through ASC protein, the pro-caspase-1 was converted into caspase-1 and then formed inflammasome, which activated pro-IL-1β and pro- IL-18 to release large amounts of IL-1β and IL-18 [4]. Stimulation of IL-1β caused the continuous activation of the NF-κB pathway and secretion of inflammatory cytokines directly or indirectly. These inflammatory cytokines could make NLRP3 inflammasome highly activated and expressed more IL-1β and IL-18, with persistent synovium inflammation and bone degradation of the joints, leading to severe joint lesions and even loss of motor function. It was reported that the effective components of PZH, ginsenoside Rg 1 [14, 38] and ginsenoside metabolite compound K [15], had an inhibitory effect on the activity of NLRP3 inflammasome. Our study further found that PZH could inhibit the activation of NLRP3 inflammasome in the joint of CIA mice.

Zinc finger protein A20, also known as tumor necrosis factor α induced protein 3 (TNFAIP3), is a key molecule that negatively regulates the inflammatory response [39, 40]. A20 exerts biological effects through the editing function of ubiquitination, which is mainly reflected in the regulation of cell differentiation and apoptosis, inflammatory response and immune regulation [41]. Previous studies indicated that A20 was an effective negative regulator of NLRP3 inflammasome and NF-κB signaling pathway [27, 42]. In RA patients, the expression of A20 in peripheral blood mononuclear cells and synovial fluid of joints was significantly lower than normal level [43]. By using CIA mice with A20 knockout, a research revealed that A20 could inhibit the activation of NLRP3 inflammasome to relief symptom [27]. Another research showed that using A20 to block the NF-κB pathway in the joints reduced both the inflammatory response and the tissue destruction in CIA mice [44]. These researches indicated that A20 was a potential target for the treatment of RA. One of the active constituents of PZH, Ginsenoside F1, has been reported to have a regulatory effect on the expression of A20 [45]. In this study, we found that the protein level of A20 was significantly decreased in the joint of CIA mice when compared with the normal mice, which was consistent with the previous study. PZH could significantly increase the expression of A20 in the joints of CIA mice. It implied that the regulatory effect of PZH on NLRP3 inflammasome and NF-κB signaling pathway might be related to promoting the expression of A20.

In this study, the efficacy of PZH on CIA mice was evaluated, and the regulation of PZH on NF-κB signaling pathway and NLRP3 inflammasome was studied. However, the research on the effect and related mechanism of PZH on bone destruction in CIA mice was lacked in this study, although alleviation of bone destruction was found by histological analysis. In addition, previous studies indicated that A20 could also alleviate bone damage in CIA mice [44]. Therefore, we will focus on A20 and its related signal pathway to further investigate the effect of PZH on bone destruction in CIA mice.

## Conclusion

In conclusion, this study preliminarily explored the effect and the possible action mechanism of PZH on CIA mice. The results showed that PZH performed a good therapeutic effect on CIA mice, and the mechanism might be related to the regulation of NF-κB signaling pathway and NLRP3 inflammasome.

## Data Availability

The datasets used and analyzed during the current study are available from the corresponding author on reasonable request.

## Abbreviations

RA: Rheumatoid arthritis
NLRP3: NOD-like receptor family pyrin domain-containing 3
NSAIDs: Non-steroidal anti-inflammatory drugs
TCM: Traditional Chinese medicines
PZH: Pien Tze Huang
CIA: Collagen-induced arthritis
MTX: Methotrexate
